# Environmental Health Workforce – Essential for Interdisciplinary Solutions to the COVID-19 Pandemic

**DOI:** 10.1017/dmp.2020.242

**Published:** 2020-07-14

**Authors:** Benjamin J. Ryan, Raymond Swienton, Curt Harris, James J. James

**Affiliations:** Baylor University, Waco, Texas; University of Texas Southwestern, Dallas, Texas; University of Georgia, Athens, Georgia; Society for Disaster Medicine and Public Health, Inc, Rockville, Maryland

**Keywords:** COVID-19, disasters, environmental health, resilience, risk management

## Abstract

Interdisciplinary public health solutions are vital for an effective coronavirus disease 2019 (COVID-19) response and recovery. However, there is often a lack of awareness and understanding of the environmental health workforce connections and capabilities. In the United States, this is a foundational function of health departments and is the second largest public health workforce. The primary role is to protect the public from exposures to environmental hazards, disasters, and disease outbreaks. More specifically, this includes addressing risks relating to sanitation, drinking water, food safety, vector control, and mass gatherings. This profession is also recognized in the Pandemic and All-Hazards Preparedness and Advancing Innovation Act of 2019. Despite this, the profession is often not considered an essential service. Rapid integration into COVID-19 activities can easily occur as most are government employees and experienced working in complex and stressful situations. This role, for example, could include working with leaders, businesses, workplaces, and churches to safely reopen, and inspections to inform, educate, and empower employers, employees, and the public on safe actions. There is now the legislative support, evidence and a window of opportunity to truly enable interdisciplinary public health solutions by mobilizing the environmental health workforce to support COVID-19 response, recovery, and resilience activities.

As the COVID-19 pandemic continues, rapid integration of the environmental health workforce to the response and recovery will enable solutions that are interdisciplinary, sustainable and focused on strengthening resilience. The environmental health workforce, which is often considered part of public health, consists of professionals who are involved in research, technology, policy, and translating and applying advances in science and technology.^1^ This last group is often used by environmental protection or public health agencies and is a foundational function of state and local governments.^2^ For example, after federal aid has been provided, standards (such as air, food, water, sanitation, and hygiene) and national policy have been set, the state and local levels of government provide environmental health services. This workforce is where services reach people, providing a great opportunity to define environmental health needs of communities and understand how these can be met.^1^

By engaging this profession, leaders across the United States would have access to the second largest public health workforce, second only to public health nursing.^3^ Their primary role is to protect the public from exposures to environmental hazards, disasters, and disease outbreaks.^2^ More specifically, this includes addressing and managing environmental and public health risks relating to: drinking water, hazardous and general waste, sanitation, food safety, communicable diseases, vector issues, and mass gatherings.^4^ The local connection with people also allows this workforce to appreciate and understand political, social, economic, cultural, and other factors that help define environmental health needs, priorities, goals, and objectives for neighborhoods or the community at large.^1^ Vital elements for maximizing the control and prevention of disease outbreaks and pandemics, such as COVID-19.

Professionals in this field working at health departments have the responsibility to address environment-related threats and the social determinants of health.^5^ This is achieved through delivery of the Centers for Disease Control and Prevention (CDC) 10 Essential Environmental Public Health Services.^6^ These include: monitoring environmental health status; diagnosing and investigating community health hazards; informing, educating, and empowering people; mobilizing community partnerships; developing plans and policies; enforcing environmental health laws and regulations; linking people with services; assuring a competent workforce; evaluating population based services; and researching/developing innovative environmental public health solutions.^6^ All are actions required for maintaining social protections, community well-being, and public health services before, during, and after disasters and pandemics.^7^

Despite these capabilities and local connections, there has often been a lack of awareness, engagement, and understanding among decision makers and leaders about the potential role of the environmental health workforce in mitigating and controlling disease outbreaks, such as COVID-19.^8^ To increase this awareness, the profession was formally recognized in the Pandemic and All-Hazards Preparedness and Advancing Innovation Act of 2019. Environmental health professionals are now included along-side physicians, nurses, and first responders. The law also directs reporting on public health preparedness and response capabilities of hospitals, long-term care facilities, and other health-care facilities to include environmental health.^8^ This will hopefully help ensure the environmental health workforce is actively involved in future strategies, committees, and workshops for mitigating, preventing and responding to disasters, emergencies, and pandemics.

The role of environmental health in COVID-19 and future pandemics would build on existing skills and tasks. This could include working with businesses and workplaces to safely reopen and developing community-based plans for continuing activities. Complementary to this there could be ad hoc visits and inspections of businesses and workplaces to inform, educate, and empower employers and employees on actions to continue safe operations. Support could also be provided to contact tracing activities (similar to the role in food-borne illnesses); collecting samples; monitoring infection control standards at hospitals, nursing homes, and healthcare facilities; assessing environmental health risks associated with modified screening practices (such as tents in front of hospitals); inspection of hygiene and sanitation standards in public areas; and liaising with schools and churches. This modification of tasks could be easily and rapidly achieved as many environmental health professionals are government employees; often have required clearances to access businesses, workplaces, schools, and hospitals; and are experienced working with the public in complex and stressful situations.

By leveraging the interdisciplinary skills of the environmental health workforce, communities will be in a better position to address the COVID-19 epidemiologic triangle (Figure 1). The triangle demonstrates the interaction of factors that contribute to an outbreak of an infectious disease, such as COVID-19, which requires an agent/pathogen, host, and environment to spread.^9^ In this triangle, 1 factor needs to be removed for the outbreak to stop. Figure 1 demonstrates the challenge this poses with multiple professions working to solve this challenge. However, the environmental health workforce can provide a much-needed interdisciplinary connector, particularly at the local level, to monitor the situation, interventions, and more broadly reduce the impact of COVID-19.

**FIGURE 1:**
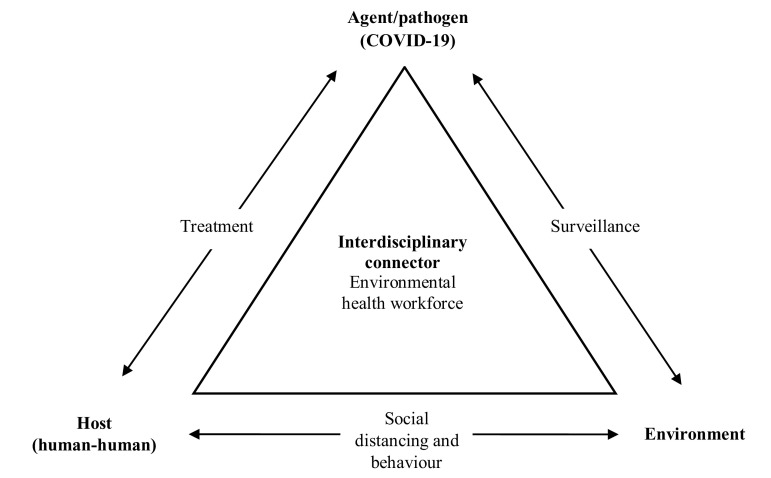
Environmental Health Role in the Epidemiologic Triangle for COVID-19.

Environmental health professionals engaged should meet certain criteria. First, it is recommended the person be a member of the National Environmental Health Association (NEHA) or a state affiliated association. This would indicate active involvement in the environmental health field. NEHA is also the peak national association and the sole United States member organization of the International Federation of Environmental Health. Second, the environmental health degree should be accredited. Ideally, this would be from a National Environmental Health Science and Protection Accreditation Council (EHAC) accredited college or equivalent if international. For example, an EHAC accredited degree, is often considered the industry standard and a requirement to enter the US Public Health Service.^9^ Third, anyone engaged should be a Registered Environmental Health Specialist/Registered Sanitarian (REHS/RS) or on the pathway to getting this credential. Applying these criteria would ensure mobilization of a qualified, skilled, and professionally supported environmental health workforce.

A mechanism to systematically and sustainably integrate this workforce could be rapid adoption of the United Nations Office for Disaster Risk Reduction’s (UNDRR) Disaster Resilience Scorecard for Public Health.^10^ This provides stakeholders, such as emergency preparedness managers, employers, and leaders, with a mechanism to collaboratively and systematically assess risk, rank and prioritize preparedness of key public health functions, infrastructure, and services for a community. This includes pandemics, health supplies chain management, vulnerable group management, surge capacity, tracking, and alerts for communities and quarantine considerations. By applying this approach, the entire spectrum of the community and disciplines involved in providing public health services would be considered, automatically integrating the environmental health workforce.

Finally, there is an urgent need for leaders and government officials at all levels to ensure their environmental health workforce is engaged in the response to COVID-19. The workforce can act as a force multiplier for the disaster cycle. The profession has, for many years, routinely prevented adverse health outcomes from disease outbreaks and disasters without wide ranging acknowledgement and awareness of their capabilities. There is now the legislative support, evidence, need, and a window of opportunity to truly enable interdisciplinary solutions to COVID-19 by mobilizing and maximizing the use of the entire spectrum of the public health workforce.
